# Commentary: Evaluation of Phase-Amplitude Coupling in Resting State Magnetoencephalographic Signals: Effect of Surrogates and Evaluation Approach

**DOI:** 10.3389/fncom.2018.00026

**Published:** 2018-04-16

**Authors:** Esther Florin, Sylvain Baillet

**Affiliations:** ^1^Medical Faculty, Institute of Clinical Neuroscience and Medical Psychology, Heinrich-Heine University, Düsseldorf, Germany; ^2^McConnell Brain Imaging Center, Montreal Neurological Institute, McGill University, Montreal, QC, Canada

**Keywords:** phase-amplitude coupling (PAC), neural oscillations, resting state, magnetoencephalography (MEG), surrogate data

## 1. Introduction

Using Human Connectome Project (HCP) data, Gohel et al. (2016) (GEA) recently reported that “resting-state MEG signals failed to exhibit ubiquitous phase-amplitude coupling (PAC) phenomenon, contrary to what has been suggested” by Florin and Baillet (2015) (FB). GEA argued that the original PAC findings by FB were driven by false positives resulting from the use of inappropriate methods. In this commentary, we first correct GEA's mischaracterization of the approach actually used by FB. We then investigated the PAC computations in Gohel et al. (2016) (GEA) and demonstrate that with FB's approach, it is actually possible to detect PAC in the Human Connectome Project (HCP) resting-state data. Finally, when making the data processing as similar as possible to GEA we still found significant PAC across large portions of the brain.

## 2. Mischaracterization of Florin and Baillet (2015)

GEA claimed that FB's significance threshold was “generated after combining all surrogate PAC scores from all frequency pairs” and argued that such pooling “of PAC scores across the frequency pairs is not appropriate,” because it would bias the finding of significant couplings toward low frequencies. This claim is incorrect: The PAC detection threshold in FB was actually computed separately for each frequency pair (page 28 in FB: “the value at the 95th quantile of PAC scores for every low/high frequency pair was determined as the significance threshold at *p* < 0.05 for actual recordings”).

## 3. Technical differences

### 3.1. Artifact removal

From GEA's statement that at least 10 independent components were removed from the data, it seems that they used their own preprocessing pipeline, not the distributed preprocessed version of the HCP data, because this latter had fewer independent components removed. After direct communication with GEA, and in an attempt to reproduce their published method, we used the preprocessed HCP data set.

### 3.2. Source modeling

GEA used a single-sphere head model, while FB used multiple-sphere forward modeling. Even though the latter is best-practice in MEG analysis (Gross et al., 2013), we tried to reproduce for the purpose of this commentary the approach of GEA by performing analyses with a single-sphere head model. However, their arbitrary selection of 58 sources where they computed PAC measures was not described with sufficient details to enable the identical reproduction of their analyses. In addition, such a small sample of sources can be expected to have biased GEA's results.

### 3.3. Frequency selection

In the FB original analysis we used all frequency pairs (*f*_φ_, *f*_*a*_), with *f*_φ_ ∈ [2, 48] Hz and *f*_*a*_ ∈ [80, 150] Hz for the calculation of PAC. The binning width for *f*_φ_ was 0.5 Hz from 2 to 12 Hz and 2 Hz from 12 to 48 Hz. The bins for *f*_*a*_ were logarithmically spaced. Following Canolty et al. (2006), we used chirplets to extract the phase and amplitude with a chirp-factor of 0, hence yielding wavelet-like decompositions.

GEA chose three low frequencies (3, 6, 10 Hz) and as high frequencies the sum over two frequency ranges, 53:10:93 and 83:10:143 Hz. To extract phase and amplitude they used Morlet wavelets with a mother wavelet of full width at half maximum (FWHM) of 1*s* and a center frequency of 1 Hz. This choice leads to 46 Hz wide FWHM filter at 53 Hz. This results in considerable smearing of the high-frequency binning.

### 3.4. Statistical detection of PAC

GEA used different statistical methods to determine statistical significance of PAC values. For the purpose of the present rebuttal, we only used one approach from GEA, which was also used in FB: We generated pink noise time series of the same lengths as the original data and calculated PAC on these surrogate data. We generated 500 pink noise time series as in GEA. For the determination of the significance threshold, GEA used a more conservative 99% quantile, while FB used 95%.

## 4. Method comparison with HCP data

We used the preprocessed data from four HCP subjects (100307, 108323, 113922, 877168). All subjects were included by GEA and we only used the one run described in the supplementary material of GEA. For preprocessing, as many ICA components were removed as described in the icaclass folder of the preprocessed data. The further analyses were conducted with Brainstorm (Tadel et al., 2011). For source reconstruction we used Fieldtrip's (Oostenveld et al., 2011) implementation to derive a single sphere head model and estimate source activations with a weighted minimum norm estimator. This is presumably the same approach as GEA used. We then computed PAC according to the method proposed by Özkurt et al. (2011) and used in FB and GEA at each of the 8,004 individual source locations.

As could be expected, using a higher significance threshold lead to fewer significant findings than in FB (Table 1). Comparing Chirplets to Morlets, fewer vertices with significant PAC were found with Morlet wavelets. Importantly the binning of low frequencies and summing of the gamma amplitudes as in GEA led to almost no PAC detection. Overall sparse sampling of the low frequencies had a greater influence on the detection of PAC than the sparse sampling in the gamma range.

**Table 1:** Average percentage of significant PAC across the brain for HCP subjects 100307, 108323, 113922, and 877168 with different frequency binning options.

	**Chirplets**		**Morlet wavelets**	
**Percentile**	**95%**	**99%**	**95%**	**99%**
**FREQUENCY BINNING ACCORDING TO FB**				
Sign. sources	100 ± 0.1	87.9 ± 5.1	37.4 ± 13.4	12.9 ± 12.3
Coupling δ/θ−γ	93.6 ± 3.3	59.2 ± 9.0	26.5 ± 17.0	10.6 ± 12.7
Coupling δ−γ	64.6 ± 17.2	34.0 ± 15.1	22.9 ± 18.9	9.9 ± 13.1
**FREQUENCY BINNING ACCORDING TO GEA**				
Sign. sources	19.3 ± 4.1	4.1 ± 2.8	10.1 ± 9.3	4.5 ± 7.7
Coupling δ/θ−γ	15.6 ± 2.4	3.4 ± 2.7	9.9 ± 9.5	4.5 ± 7.7
Coupling δ−γ	10.6 ± 2.5	2.5 ± 2.0	8.1 ± 10.6	4.3 ± 7.8
**NO SUMMING IN GAMMA FREQUENCY RANGE; LF ACCORDING TO GEA**				
Sign. sources	68.6 ± 7.7	21.4 ± 9.4	13.9 ± 9.4	5.4 ± 8.4
Coupling δ/θ−γ	55.5 ± 9.9	15.4 ± 9.2	13.2 ± 9.6	5.4 ± 8.4
Coupling δ−γ	37.3 ± 6.8	9.8 ± 6.7	9.8 ± 11.6	5.0 ± 8.6
**LF FROM 3 TO 10 Hz WITH 0.5 HZ SPACING; HF ACCORDING TO GEA**				
sign. sources	74.6 ± 8.4	32.0 ± 15.2	21.7 ± 14.5	9.0 ± 12.8
coupling δ/θ−γ	66.6 ± 8.5	28.3 ± 16.6	21.3 ± 14.6	9.0 ± 12.8
coupling δ−γ	35.0 ± 5.6	7.9 ± 2.2	18.0 ± 16.4	8.8 ± 13.0

*HF, high frequencies included in the PAC analysis; δ, 2–4 Hz; θ, 4–8 Hz; γ, for FB 80–150 Hz and for GEA 53–143 Hz; δ/θ−γ, significant PAC when restricting the low frequencies to δ/θ frequencies; δ−γ, significant PAC when restricting to δ frequencies*.

## 5. Discussion

We investigated the claim from GEA that the results of FB were only due to false positives. We did so by using four of the HCP datasets used by GEA, staying as close as possible to their approach.

Using the sparse low-frequency binning of GEA led to pronounced reduction in PAC detection. Thus, it is likely that the actual low-frequency phase component was missed using GEA's methodology. We believe that not restricting PAC analysis to few frequencies pairs provides a more encompassing picture of potential oscillatory coupling in the brain.

We used pink (1/*f*) Gaussian noise for the determination of the significance threshold as in FB. GEA demonstrated that using pink Gaussian noise is less conservative than the non-colored noise approach. While this might be the case, pink noise is the preferred option, because neural signals exhibit a 1/*f* frequency spectrum and non-parametric statistical testing requires that one should target the original data properties of non inferential interest with surrogate data. Of note for future research is that the non-sinusoidal shape of brain oscillations might affect the detection of cross-frequency coupling depending on the method used to extract the phase and amplitude coupling data (Yeh et al., 2016; Cole and Voytek, 2017). In principle, chirplets and wavelets can be constructed to account for the expected non-sinusoidal shape of brain oscillations (Mallat, 2009).

In summary, we can only restate that ubiquitous PAC in resting state MEG data is present across the brain. This was also the case for data from the HCP repository if all potential low-frequency high-frequency pairs are tested exhaustively.

## Ethics statment

We used publicly available, open access data from the human connectome project.

## Author contributions

All authors listed, have made substantial, direct and intellectual contribution to the work, and approved it for publication.

### Conflict of interest statement

The authors declare that the research was conducted in the absence of any commercial or financial relationships that could be construed as a potential conflict of interest.
